# rs4889 polymorphism in *KISS1* gene, its effect on polycystic ovary syndrome development and anthropometric and hormonal parameters in Saudi women

**DOI:** 10.1186/s12929-018-0452-2

**Published:** 2018-05-30

**Authors:** Fadwa S. Albalawi, Maha H. Daghestani, Mazin H. Daghestani, Abdelmoneim Eldali, Arjumand S. Warsy

**Affiliations:** 1 Department of Zoology, Center for Scientific and Medical Female Colleges, King Saud University, P.O. Box 22455, Riyadh, 11495 Saudi Arabia; 2 Department of Obstetrics & Gynecology, Umm-Al-Qura University, P.O. Box 424, Makkah, 21955 Saudi Arabia; 3 Department of Biostatistics, Epidemiology &Scientific Computing, King Faisal Specialist Hospital and Research Center, P.O. Box 3354, Riyadh, 11211 Saudi Arabia; 4 Central Laboratory, Center for Scientific and Medical Female Colleges, King Saud University, P.O. Box 22455, Riyadh, 11495 Saudi Arabia

**Keywords:** *KISS1* gene, Kisspeptin, LH, FSH, GPR54, PCOS

## Abstract

**Background:**

Kisspeptin is involved in female reproduction. This study was designed to i- estimate kisspeptin levels in women with polycystic ovary syndrome (PCOS), in comparison with controls, ii- study the correlations between kisspeptin and PCOS-related reproductive hormones, and iii- investigate the relation between *KISS1* gene polymorphisms and hormone levels in women suffering from PCOS.

**Methods:**

The investigation was a clinically designed study on 28 women with PCOS, and 30 normal, healthy women with no signs of PCOS as controls. Blood samples were collected between day 3 and day 6 of the menstrual cycle in both groups at 8:00 a.m., and circulating levels of LH, FSH and kisspeptin were estimated. DNA was extracted from whole blood and all coding exons of *KISS1* gene were sequenced.

**Results:**

Women with PCOS had higher LH levels and BMI compared to controls. Plasma kisspeptin levels were positively correlated with LH levels. There was no statistically significant difference between the groups in terms of kisspeptin and FSH levels. The SNP rs4889 C/G, a non-synonymous SNP, was investigated in the PCOS group. The frequency of GG genotype was significantly higher in the PCOS compared to the controls. These patients were more obese, had higher kisspeptin and FSH levels.

**Conclusion:**

The results of the study show that the genetic variation of *KISS1* gene may be a factor contributing to PCOS development. The association between the gene and the gene variation and PCOS need further validation in large-scaled and functional studies.

## Background

Kisspeptin is a 54-amino-acid peptide encoded by the *KISS1* gene, and is also known as metastin [1, 2], which was first isolated from the human placenta in 2001 [3]. Kisspeptin action is exerted by a *trans*-membrane G-protein coupled receptor, named GPR54, AXOR12, or HoT7T175 [4]. Loss of *KISS1* gene function is reported to be associated with hypogonadotropic hypogonadism in humans and animal models [5, 6]. Binding of kisspeptin to its receptor (GPR54) in the GnRH neurons in the hypothalamus, results in the stimulation of gonadotropin release, which in turn binds to the GnRH receptors in the pituitary and influences the release of LH and FHS [7]. These hormones act on the gonads and influence estrogen, testosterone and progesterone release. Hence, kisspeptin has been shown to regulate the secretion of luteinizing hormone (LH) during the promotion of ovulation [8] by stimulating gonadotropin releasing hormone (GnRH) from the hypothalamus [9, 10].

The polycystic ovary syndrome (PCOS) is a common heterogeneous disorder affecting women, with a prevalence of 6–12% in women of reproductive age [11]. It is of complex pathogenesis, characterized by hypothalamic-pituitary disturbances in gonadotropin secretion, specifically increased LH levels [12, 13], chronic anovulation, and polycystic ovaries (PCO) on ultrasound. In addition, patients frequently suffer from hyperinsulinemia, insulin resistance [14], type 2 diabetes mellitus (DM), cardio-vascular disease and infertility [15, 16]. Given the complex relationship between kisspeptin and hypothalamic-pituitary gonadal axis, the present study aimed to investigate the relation between *KISS1,* BMI, FSH and LH and the influence of *KISS1* gene polymorphisms on these parameters in normal women and those suffering from PCOS.

## Methods

### Subjects

The samples were collected from Al-Hira Hospital in Makkah Al Mukarama, from 58 Saudi women volunteers aged 19–36 after obtaining Ethical Committee approval, from the Institutional Review Board. Each participant gave a written informed consent prior to enrolment in the study. A total of 58 Saudi women volunteers aged 19–36 years were recruited. Cases were 28 Saudi women attending the outpatient clinics and fulfilling the following criteria:

- ○ Oligomenorrhoea (cycle duration between 35 days to 3 months).
- ○ Transvaginal ultrasound image of enlarged ovaries with > 10 cysts in the largest plane; each measuring <I0mm in diameter scattered around an echodense thickened stroma [17]
- ○ None had received any drugs known to interfere with hormonal concentrations for at least 3 months before the study.

Controls were 30 Saudi women volunteers representing cross section of Saudi society. All subjects were healthy, had spontaneous onset of puberty and sexual development, with regular menstrual cycle, and no history of gastrointestinal or endocrine disorders.

### Anthropometric measurements

Anthropometric measurements included body weight, height and body mass index (BMI), (weight in Kg divided by height in m^2^).

### Laboratory methods

Blood samples were collected between day 3 and day 6 of a menstrual cycle in the PCOS and control groups, all blood samples were obtained at 8:00 a.m. (This ensured obtaining the true level of the reproductive hormones, as these hormones show variations with the days of the menstrual cycle). Five milliliter of blood was drawn by venipuncture in red top tube for kisspeptin, FSH and LH estimations.

In addition, 5 mL of blood was collected in EDTA-coated tubes for DNA extraction. All blood samples for each subject were immediately centrifuged, and plasma, serum and buffy coat were stored at − 80 °C until analysis. Kisspeptin levels were measured with an enzyme-linked immunoassay kit (ELISA kit, Phoenix Pharmaceuticals Inc., Belmond, CA), after extraction with Phoenix Peptide sep-columns (RK-Sepcol-2). FSH and LH levels were measured with an enzyme-linked immunoassay kit (ELISA kit, Human, cat.No.65205.GER).

### Genotyping of *KISS1* gene polymorphism

Genomic DNA was extracted from peripheral blood leucocytes using commercially available Puregene Blood Kit (QIAGEN, cat. No. 158389, USA). Polymorphism was determined by sequencing following polymerase chain reaction (PCR), using forward and reverse primers. The primers were designed using PRIMER 3 program and the sequence of each primer was as follows:

F: 5′- ACCTGCCGAACTACAACTGG-3′; R: 5’-TGAAGGAACAGGCGGTTAGT -3’.

The PCR conditions consisted of initial denaturation step at 95 °C for 15 min, followed by 34 cycles of denaturation at 95 °C for 1 min, annealing at 60 °C for 1 min, and extension at 72 °C for 1 min, with final extension of 10 min at 72 °C. A PCR product of 353 bp was obtained. Nucleotide sequencing was carried out using the ABI Big Dye Terminator protocol on ABI 3100 Avant Genetic Analyzer.

## Statistical analyses

The descriptive characteristics of the group variables were expressed as mean ± SEM. The comparisons between PCOS patients and their matched controls were carried out using the independent t-test and ANOVA test with respect to all variables. Pearson Correlation Coefficients was used to study the correlation between Kisspeptin, BMI and other studied variables. Genotype and allele frequencies were calculated manually https://ihg.gnf.de/cgi-bin/hw/hwa1.pl. Significance of the difference in the result of POCS cases and controls was obtained using Fisher’s Exact test (two-tailed) and odds ratios, 95% confidence intervals, χ2 and *p* value were obtained. All statistical analyses were performed by using SPSS for Windows (version 9.3).

## Results

Basic anthropometrics and hormonal features of the study group are summarized in Table 1. Women with PCOS had significantly higher weight, BMI and LH level compared to the control group (*p* < .0001). No significant differences in FSH and kisspeptin level between groups were observed. Kisspeptin level were higher in PCOS women compared to control group, but not significantly. Kisspeptin level were found to be positively correlated with LH level in the PCOS patients (*r* = 0.604; *p* = 0,005, control: *r* = 0.409; *p* = 0.007). Correlations between kisspeptin level, hormonal and anthropometric measurement of women studied are summarized in Table 2. Direct sequencing of *KISS1* gene revealed several SNPs. The SNP rs4889 (C/G) was detected more frequently in PCOS group than in control, and this nonsynonymous SNP results in the substitution of P81R. Distribution of rs4889 C/G in *KISS1* gene and the allele and genotype frequencies in PCOS and control groups are summarized in Table 3. Statistically significant differences were observed for the alleles (*p* = 0.0057), and genotype frequency between PCOS and controls. Allele C showed higher frequency in PCOS group (37.5%) than control group (15%). The individuals with the different genotypes were separated and the levels of the anthropometric and hormonal parameters and kisspeptin were separately analyzed for each genotype. Table 4 presents the results of the study variables in the different rs4889 genotypes (CC, CG and GG) in Saudi women with PCOS and Control groups. Statistically significant difference were observed in the wild type GG in women with PCOS in weight (Kg) (75.83 ± 11.42vs. 57.59 ± 10.07, *p* < .0001), and in BMI (wt/ht^2^) (48.32 ± 6.73 vs 35.01 ± 8.04, *p* < .0001). Statistically significant difference were observed in the heterozygous CG in LH level (13.73 ± 3.35 vs. 8.741 ± 1.28, *P* < .0001). The effect of the different genotypes of rs4889 on the level of of kisspeptin was studied and as shown in the Table 4. The kisspeptin level did not differ significantly in individuals with the CC, CG and GG genotypes in PCOS group and GG and CG in control group. Only one control had CC genotype and was not included in the calculations.

**Table 1:** Comparisons of clinical parameters amongst PCOS and control group

Variable	PCOS Group (*n* = 28) Mean ± SD))	Control Group (*n* = 30) Mean ± SD))	*P*-value
Age (years)	29.4 ± 3.93	26.7 ± 3.6	0.008
Weight (Kg)	72.63 ± 12.8	59.87 ± 10.8	0.0001
Height (cm)	155.9 ± 6.9	161.5 ± 4.1	0.001
BMI (Kg\m^2^)	46.04 ± 7.3	36.6 ± 8.17	0.0001
FSH (IU\I)	6.81 ± 5.4	5.7 ± 1.95	0.325
LH (IU\I)	15.12 ± 3.7	8.73 ± 1.16	0.0001
Kisspeptin (pg\mL)	0.43 ± .15	0.39 ± .07	0.178

**Table 2:** Correlations between kisspeptin level, hormonal and anthropometric measurement of PCOS patients and control group

Correlation between kisspeptin and	PCOS Group (*n* = 28)			Controls Group (*n* = 30)		
	*r*	*p*	Sig	*r*	*P*	Sig
Age (years)	0.182	0.355	NS	0.083	0.663	NS
Weight (Kg)	0.111	0.575	NS	0.152	0.422	NS
Height (cm)	−0.118	0.549	NS	−0.177	0.351	NS
BMI (Kg/m^2^)	0.165	0.401	NS	0.109	0.567	NS
FSH (IU/I)	0.049	0.804	NS	0.225	0.231	NS
LH (IU/I)	0.604	0.005	S	0.409	0.007	S

*NS* non-significant = *p* > 0.05, *S* significant = *p* ≤ 0.05, *r* correlation coefficient

**Table 3:** Frequencies of allele and genotypes of rs4889 C/G in *KISS1* gene in PCOS and control groups

Variation	Control (*N* = 30)	Case (28)	OR	CI	χ ^2^	*P*-value
CC	1 (3.3)	4 (14.3)	8.0	0.79–80.4	3.96	0.046
CG	7 (23.3)	13 (46.4)	3.714	1.15–11.96	5.04	0.024
GG	22 (73.3)	11 (39.4)	0.125	0.012–1.26	3.96	046
CG + GG	29 (0.48)	24 (0.43)	0.207	0.02–1.98	2.21	0.137
C	9 (0.15)	21 (0.375)	3.4	1.4–8.3	7.65	0.006
G	51 (0.85)	35 (0.625)	0.294	0.12–0.72	7.65	0.006

*OR* odds ratio, *CI* confidence intervals, *χ*^*2*^ chi square, *P* significance

**Table 4:** Level of anthropometric parameters and hormones in different genotypes of rs4889 C/G in *KISS1* gene in Saudi women with PCOS and Control groups

Variable	PCOS Group (*n* = 28) (Mean ± SD)			Control Group (*n* = 30) (Mean ± SD)		*P*-value		
	GG *n* = 11	CG *n* = 13	CC *n* = 4	GG *n* = 22	CG *n* = 7	GG	CG	CC
Weight (Kg)	75.8 ± 11	74.1 ± 13	58.8 ± 4.1	57.5 ± 10	68.4 ± 9.6	0.0001^**^	0.362	0.157
Height (cm)	156. ± 6.8	156 ± 7.5	152 ± 5.91	162 ± 3.9	158.8 ± 3.5	0.007^*^	0.660	0.468
BMI (Kg/m^2^)	48.3 ± 6.7	47.2 ± 7.5	38 ± 1.76	35 ± 8	42.6 ± 6.43	0.0001^**^	0.219	0.157
FSH (IU/I)	6.52 ± 5.6	7.64 ± 5.8	4.9 ± 4.02	5.8 ± 2	5.5 ± 1.4	0.879	0.475	1.00
LH (IU/I)	13.7 ± 3.3	16.2 ± 3.8	15.3 ± 3.7	8.7 ± 1.2	8.6 ± 0.89	0.0001^**^	0.0001^**^	0.147
kisspeptin (pg\mL)	0.38 ± 0.2	0.43 ± 0.1	0.35 ± 0.18	0.37 ± .06	0.45 ± 0.06	0.349	0.968	1.00

There was only 1 sample with a CC genotype in the control group, and was not used in the comparison

^*^p-value< 0.05; ^**^p-value< 0.001

## Discussion

The present study investigated plasma kisspeptin levels in Saudi women with and without PCOS and studied the nature of correlation between kisspeptin, anthropometric parameters and PCOS-related reproductive hormones. It also evaluated the sequence of exons in the *KISS-1* gene and compared the frequency of rs4889 C/G, a non-synonymous SNP, in PCOS and controls. Our investigation showed that the level of kisspentin was slightly higher in females with PCOS, though the results compared to the control group were not significantly different. Three previous studies [18–20] reported higher kisspeptin levels in women with PCOS. Another study [21] reported lower levels in women with PCOS as compared to controls. Yerlikaya et al., [22] study and our study did not support these findings. This may be the result of the obesity and insulin resistance that may have negative impact on kisspeptin levels.

The Kiss-1 system has emerged in the recent years as a fundamental player in the control of the reproductive axis, with essential roles in differentiation and pubertal activation of the reproductive system as well as key functions in the regulation of ovulation and fertility [18]. Kisspeptin has been recently associated with increased GnRH and regulates the secretion of LH during the promotion of ovulation [23, 24]. PCOS is a condition associated with disordered hypothalamic-pituitary-gonadal axis, higher LH levels, compared to ovulatory women without the syndrome [25, 26]. The present study evaluated the possible role of kisspeptin in the pathophysiology of PCOS and showed the correlation between levels of kisspeptin and LH. Several groups have now reported that kisspeptin administered either centrally or peripherally, stimulate gonadotropin secretion. Similar observations were reported in the rat [27, 28], sheep [29], monkey [30, 31] and more recently, the human male [32]. Our study showed that LH levels were higher in PCOS women compared to controls, and plasma kisspeptin levels were positively correlated with LH levels. It was consistent with the idea that kisspeptin may stimulate LH secretion, although the way of direct pituitary effects of kisspeptin in the control of gonadotropin secretion remains controversial [33, 34]. In another study conducted by Panidis et al., [21], it was found that LH levels were significantly higher in PCOS groups compared to controls. This finding was in line with the results of the present study. However, no significant correlation between plasma kisspeptin and LH levels was observed in their study, and they also found that obese and overweight women with PCOS had significantly lower kisspeptin levels compared to normal weight women with the syndrome. These conclusions are contradictory to our study, possibly due to differences in the BMI in these studies as well as methodological differences. Additionally, small sample size and the heterogeneity of the study groups are the limitations of these previous studies. Plasma kisspeptin levels were measured by Chen et al. [19] in 42 women with PCOS (23 adult, 19 adolescents) and 20 adolescent controls to investigate the possible correlations between kisspeptin and PCOS related reproductive and metabolic disturbances. Their results suggested that plasma kisspeptin levels were increased in lean adolescent and adult women with PCOS compared with lean adolescent control group. Our study did not support these findings. Moreover, LH levels of both adult and adolescent PCOS women were higher than that of adolescent controls and kisspeptin showed a positive correlation with LH similar to the findings of our study. Some researchers have demonstrated that kisspeptin may play a key role in the activation of the gonadotropic axis at puberty [35, 36]. So elevated kisspeptin levels of adolescent PCOS may play a role as a marker to recognize PCOS in adolescents more clearly and sometimes at an earlier stage. In the study of Chen and coworkers [19], the number of the controls was relatively small and no adult controls were involved. Therefore, it needs further research to draw any conclusions. Plasma kisspeptin levels were measured by Jeon et al. [20] in 54 women with PCOS and 36 controls. Their results suggested that plasma kisspeptin levels were significantly higher in the PCOS group than in controls; however, they did not find correlations between kisspeptin and any of the hormones. The data of Yilmaz et al., [18] claimed that women with PCOS exhibited higher kisspeptin levels than controls. Furthermore, kisspeptin concentrations were found to be in a positive correlation with LH levels.

In this study, polymorphism in SNP rs4889 C/G was detected more frequently in PCOS group than in control (*p-value* = 0.0057). Allele C showed higher frequeny in PCOS group (37.5%) than control group (15%). When the results in different genotypes were compared, the weight and BMI were higher, while the LH level was lower in the GG genotype, in the PCOS patients and the results were significantly different. Kisspeptin was higher in the GG genotype but the results were not significantly different. When compared with the control group, the weight, height and BMI were significantly higher in PCOS carrying GG genotype, compared to the controls with the same genotype. LH was higher in all genotypes in the PCOS, compared to the results in the control group.

rs4889 polymorphism introduced a substitution of proline for arginine at the 81 position. This is a substitution that was observed in kisspeptin-54, but not in the other three forms of kisspeptin (kisspeptin-14, − 13, and − 10). This SNP is found in the coding region and results in the substitution of an imino acid Pro by a basic amino acid Arg. Change in the DNA sequence as a result of this polymorphisms could alter the structure, function and binding capacity of kisspeptin to its receptor GPR54.. Previous studies in Korean and Chinese populations found no relationship between this SNP and CPP [37, 38]. From the results of this study, it appears that rs4889 influences the mechanism by which kisspeptin activates secretion of LH but not FSH. Further studies are necessary to investigate the possible mechanisms involved in kisspeptin function and mode of action.

## Conclusion

In conclusion, this study has shown significant relationship between the rs4889 C/G polymorphisms in KISS1 gene and PCOS in Saudi females, where the mutant G allele is highly protective (OR = 0.294; 95% CI = 0.12–0.72, χ2 = 7.65; *p* = 0.006). The LH levels were higher in the women with PCOS, compared to the controls, and plasma kisspeptin levels positively correlated with LH levels. Within the genotypes of rs4889 the LH levels were significantly lower in the GG compared to the CG and CC genotypes in the PCOS females. The mechanism of kisspeptin for regulating gonadotropin secretion remains unknown. Discrepant findings among the results of the published studies may be attributed to the design and sample size and the demographic and genetic characteristics of the different populations.

The major limitation of our study was the small number of the studied sample. Further studies are warranted, to get a clearer picture of the mechanisms that bring about the interaction between kiss-1 gene and different hormonal parameters.
